# Canine Tooth Microbiome Gingival Index: a new microbiome-derived measure of gingival health validated by nutritional intervention

**DOI:** 10.3389/fvets.2026.1839039

**Published:** 2026-06-05

**Authors:** Regina Hollar, Chun-Yen Cochrane, Nicole Green, Lori Coffman, Dale Scherl, Dayakar V. Badri

**Affiliations:** Science and Technology Center, Hill’s Pet Nutrition, Inc., Topeka, KS, United States

**Keywords:** canine, Gingival Index, nutritional intervention, oral microbiome, subgingival plaque

## Abstract

**Introduction:**

Periodontitis affects over 80% of dogs over 3 years of age, progressing irreversibly from gingivitis due to an imbalance in the subgingival microbial community that triggers an immune response. Early diagnosis of gingivitis is challenging, often relying on visible redness or bleeding noticed by pet owners or professionals. Therefore, an easy-to-interpret, clinically relevant, and responsive measurement tool based on the subgingival microbiome is needed to facilitate early diagnosis of oral health issues. We developed the Canine Tooth Microbiome Gingival Index (CTMGI), a single-score metric derived from subgingival plaque microbiome data and machine learning models, and validated its responsiveness *via* nutritional intervention.

**Methods:**

We collected subgingival plaque microbiome profiles from 692 tooth samples of 347 dogs, generated through 16S amplicon sequencing. For the machine learning models, the tooth gingivitis scores were dichotomized into healthy (gingivitis score <3) and unhealthy (gingivitis score ≥3), along with other clinical scores such as tooth recession, pocket depth, and attachment loss. The raw data were split into training and test sets, and five distinct machine learning models were employed to identify features that distinguish healthy from gingivitis sites.

**Results:**

The two top-performing models—random forest and logistic regression—yielded 22 unique features. These 22 features included the sum of early and late colonizers, the phyla actinobacteria and proteobacteria, and other bacterial species. The CTMGI was derived from the 22 features, categorized as “positive” or “negative” based on their influence on gingivitis. The CTMGI classification cutoff score was set at −0.12 with a Receiver Operating Characteristics-Area Under the Curve (ROC-AUC) of 0.761, a sensitivity of 0.701, and a specificity of 0.752. A score greater than −0.12 was found to indicate a “healthy” gingival condition; otherwise, it indicated “unhealthy.” Furthermore, we conducted a nutritional intervention study to validate the responsiveness of the CTMGI, in which the test food, which has documented oral health benefits, resulted in a significantly higher CTMGI score (1.32) compared to the control food, which offers no oral health benefits (0.66).

**Discussion:**

Overall, this study developed and validated a quantitative, single-score, microbiome-based metric that is clinically translatable for the assessment of early-stage canine gingival health. Furthermore, its demonstrated responsiveness to nutritional intervention suggests that this index can serve as a prognostic measure.

## 1 Introduction

Affecting up to 80% of dogs over 3 years of age, periodontal disease is a highly prevalent condition that encompasses both gingivitis and periodontitis, making it one of the most common diagnoses in veterinary medicine (1–5). Smaller breeds face an elevated risk due to factors such as tooth crowding, which facilitates plaque retention, with extra-small breeds (<6.5 kg) being five times more likely to develop the disease than giant breeds (>25 kg) (6–9). The effort to understand this widespread condition has spurred an evolution in thought, beginning with the “non-specific plaque hypothesis,” which suggested that the entire plaque biofilm was pathogenic (10). This view shifted with the “specific plaque hypothesis,” which identified key pathogenic species (11), and later evolved into the “ecological plaque hypothesis,” which proposed that environmental disruptions favor disease-causing microbes (12). Later, the “keystone pathogen hypothesis” was proposed, which suggested that certain low-abundance pathogens can orchestrate a shift to a dysbiotic state (13). Most recently, the “IMPEDE” model has emerged, highlighting the host’s immune response as critical in maintaining microbial balance. This model suggested that a failure of this system allows pathogens to dominate and drive disease progression (14). Collectively, these hypotheses underscore the critical interplay between plaque composition and host immunity in the development of periodontal disease.

The canine oral microbiome is a complex ecosystem composed of stable commensal species and a variable community that is essential for maintaining periodontal health through physiological and immune support (15). This delicate balance is shaped by environmental, lifestyle, and genetic factors, but it is highly susceptible to disturbances. When the balance is disrupted, the community can undergo a dysbiotic shift—not a simple replacement of “good” bacteria with “bad,” but a complex change across the entire microbial network. In this altered state, pathogenic consortia, such as the “red complex” bacteria including *Porphyromonas gulae*, can emerge and thrive in an inflammatory environment, driving tissue destruction (16, 17).

Recent research using clinical surveys and next-generation sequencing technologies has significantly advanced our understanding of the canine oral microbiome’s diversity, dynamics, and associations with disease (18–20). Studies have identified hundreds of taxa linked to either health or disease, demonstrating that specific pathogenic species such as *Porphyromonas*, *Fusobacterium*, and *Treponema* are strongly implicated in disease onset, biofilm development, and the shift toward more anaerobic, pathogenic communities (20–22). Age, breed, hygiene, diet, and other environmental factors further modulate risk (7, 23–26); however, direct studies tracking longitudinal microbiome changes alongside clinical progression are still rare (27). Although advances in targeted molecular diagnostics have improved detection and risk assessment (5, 28), inconsistencies in methods and sampling protocols, particularly regarding oral site specificity, continue to limit broader insight.

A deep understanding of the canine oral microbiome requires distinguishing among its ecological niches—oral mucosa, tongue dorsum, saliva, and both supra- and subgingival plaque—each supporting distinct microbial communities (17, 18, 20, 29, 30). Of these, subgingival plaque, which develops within anaerobic periodontal pockets, has the strongest association with the pathogenesis of periodontitis (14, 18). Saliva, while easy to sample, only partially reflects the communities found in more relevant niches, such as plaque and mucosal surfaces (17, 18, 31), reinforcing the necessity of site-specific sampling for robust microbiome-disease correlation.

Food plays a pivotal role in shaping the composition and function of the oral microbiome in companion animals, directly influencing their oral health. The texture of food has a significant impact; abrasive diets and dental chews can help mechanically remove plaque, altering the microbial habitat and discouraging the establishment of pathogenic biofilms (25). Furthermore, emerging research suggests that specific dietary components can modulate the oral microbiome. For example, supplementation with certain ingredients may inhibit the growth of key periodontal pathogens such as *Porphyromonas gulae* in dogs (32). Therefore, dietary choices, including macronutrient composition and food texture, are significant modifiable factors in maintaining a healthy oral microbiome and attenuating common dental diseases in companion animals.

This study centers on the canine subgingival plaque microbiome due to its crucial role in the etiology, risk stratification, and development of interventions for periodontal disease. Our objectives are threefold: (1) characterization of subgingival plaque microbiomes in a large, diverse canine cohort using next-generation sequencing technology and correlating profiles with clinical gingivitis scores; (2) development of the Canine Tooth Microbiome gingival Index (CTMGI) as a diagnostic and monitoring tool; and (3) real-time validation of the CTMGI tool through a controlled dietary intervention study impacting subgingival microbiome composition and gingival health.

## 2 Materials and methods

### 2.1 Study 1-Cross-sectional study to profile the subgingival microbiome and develop the CTMGI

#### 2.1.1 Study design and metadata collection

This study commenced in January of 2019 and ended in December of 2019, during which subgingival plaque samples were collected from dogs in the colony located at Hill’s Pet Nutrition Center (Topeka, KS) during their annual examinations by following the standard operating procedures approved by the Hill’s Institutional Animal Care and Use committee (IACUC) and the Hill’s Animal Welfare Committee in accordance with the guide for the care and use of laboratory animals from the US National Research Council (33). These annual examinations include a physical examination, blood CBC and chemistry analyses, and a comprehensive oral health assessment and treatment (COHAT). Dental grading was performed by a single, well-trained dental grader to prevent inter-observer variability. A comprehensive examination of each dog’s oral cavity was conducted by a dental grader to assess substrate accumulation. This evaluation provided a thorough understanding of each animal’s oral health status to grade the whole mouth’s plaque, calculus, and gingivitis scores across the entirety of the oral cavity. In addition, specific tooth gingivitis scores were recorded at the sites where the subgingival plaque samples were collected for each dog. Furthermore, intra-oral data collected from the entirety of the oral cavity of each dog encompassed the following clinical parameters: recession scores, gingival pocket depth scores, and attachment loss scores. The age of each dog, the time between the last and current dental prophylaxis, the dog’s disease condition, and the number of different foods fed 10 days before the dental prophylaxis were also recorded.

#### 2.1.2 Dental grading

Whole-mouth plaque, calculus, and gingivitis grading were performed as follows:

Whole mouth plaque scores with eosin staining were graded as light, medium, or heavy according to the following criteria: Light: little to no coverage and light intensity on all teeth; Medium: moderate to full coverage and moderate intensity on all teeth; and Heavy: moderate to full coverage and heavy intensity on all teeth. The severity of calculus was graded into three distinct groups: light, medium, or heavy. Light: minimal to no observable calculus on all teeth; Medium: moderate calculus on at least half to the majority of teeth; and Heavy: substantial to extensive accumulation of calculus on at least half to the majority of teeth. Whole mouth gingivitis scores were graded as light, medium, or heavy according to the following criteria: Light: minimal to no inflammation, no bleeding when probing on the majority of gingival surfaces; Medium: moderate inflammation (moderate redness), bleeding when probing on the majority of all gingival surfaces; and Heavy: severe inflammation (marked redness, hypertrophy, and/or ulceration, immediate bleeding when probing on the majority of all gingival surfaces). Individual tooth gingivitis scores were measured, followed by a modified Loe and Silness (34) scoring method. Briefly, this scoring method was performed on the mesial, buccal, and distal portions of a given tooth according to the following metrics: 0 = Normal gingiva, 0.5 = Mild inflammation with slight redness, 1 = Moderate inflammation and redness, but no bleeding on probing, 2 = Moderate inflammation with severe redness and bleeding on probing, and 3 = Severe inflammation and redness, edema, ulceration, and spontaneous bleeding. Finally, the scores were summed to represent the individual tooth gingivitis score on a scale of 0–9. Other clinical parameters, such as tooth recession (the measurement of tooth root exposure caused by the loss of gum tissue and/or the retraction of the gingival margin from the crown, measured in mm), pockets (the measurement of the pathologically deepened gingival sulcus, occurring secondary to the coronal movement of the gingival margin and the apical movement of the gingival attachment, measured in mm), and attachment loss (the measurement of the pathological detachment of collagen fibers from the cemental surface, with concomitant apical migration of the junctional or pocket epithelium onto the root surface, measured in mm). Tooth recession, pocket, and attachment loss scores <3 were considered to indicate healthy gums, and scores ≥3 were considered to be clinically concerning.

#### 2.1.3 Subgingival plaque sample collections

Two subgingival plaque samples were collected from each dog. One subgingival plaque sample was collected from an unhealthy tooth (T1), and another one was collected from a healthy tooth (T2). The unhealthy and healthy teeth were selected primarily based on the individual tooth’s gingivitis score from the entirety of the oral cavity for a given dog. If a dog did not have an unhealthy tooth, then we collected subgingival plaque samples from two different healthy teeth, and *vice versa.* Best efforts were made to collect subgingival plaque samples from the same tooth location across the dogs for consistency (Supplementary Figure S1). If a dog was missing the required tooth, another location was considered. However, any tooth considered for subgingival plaque sample collection was restricted to the so-called Veterinary Oral Health Council (VOHC) set, due to the inclusion of these teeth in whole-mouth substrate grading. Prior to plaque collection, the exposed tooth surface was meticulously cleaned with a 4 × 4 gauze pad to remove contaminants. Plaque samples were acquired by inserting a dental absorbent point (Absorbent point # 504, Henry Schein, Melville, NY, USA) into the subgingival space of the target tooth and swiping to collect the plaque. Each absorbent point, now with subgingival plaque, was placed into a sterile cryovial, immediately flash-frozen in liquid nitrogen, and subsequently stored at −80 °C until further analysis. This protocol ensured that the samples remained uncontaminated and preserved the integrity of the microbial communities, thus facilitating accurate subsequent analysis.

### 2.2 Study 2-AdHOC feeding study with nutritional intervention

#### 2.2.1 Animals, foods, and experimental design

The study protocol (FP891b.1.5.0-A-C-MULTI-DEN-MULTI-28-ORL) was reviewed and approved by the Hill’s Institutional Animal Care and Use Committee (IACUC) and by the Hill’s Animal Welfare Committee, in accordance with the guide for the care and use of laboratory animals from the US National Research Council (33). This study was performed at the Ontario Nutrition Lab, Ontario, Canada. A total of 40 clinically healthy small-breed adult dogs, identified based on their blood CBC/serum chemistry and urinalysis, were recruited for this study. No restrictions on gender or reproductive status were included, and the dogs’ ages ranged from 1.5 to 7.4 years. The adult small-breed dogs in this dietary intervention study cohort were different from those used to develop the CTMGI, as the latter were derived from a separate, larger cohort. This feeding study was performed by following a two-arm parallel design, with all 40 dogs assigned to one of two groups (*n* = 20 each) based on their gender, age, and history of whole-mouth plaque scores. All dogs underwent full-mouth dental prophylaxis (supra- and subgingival on the buccal and lingual sides), and the removal of supra- and subgingival plaque was confirmed with the use of eosin dye or a dental flashlight during prophylaxis. After dental prophylaxis, one group was fed a control diet (Purina Dog Chow complete adult, Lot # 126782), and another group was fed a test diet (Hill’s Dental Care t/d Small Bites dry, Lot # 126833) for a period of 28 days. Each dog was fed an amount of food required to maintain their body weight, calculated based on their daily caloric requirements. All dogs were permitted normal socialization and enrichment activities, which included daily group exercise and interacting with other dogs, except that they were not allowed to have chew toys or any treats.

#### 2.2.2 Whole-mouth grading

Dogs were graded under anesthesia using the Logan-Boyce method (35) for dental plaque and using the Warrick-Gorrel (36) method for calculus on day 28. Whole-mouth plaque and calculus accumulation were quantified using the VOHC (Veterinary Oral Health Council) metrics, along with those outlined below, which were applied to the “whole tooth” (no splitting for plaque and calculus). Each tooth’s dental plaque was scored based on the coverage and the thickness (eosin stain intensity) using the following numerical scale: Coverage (0 = no plaque detected, 1 = 1–24%, 2 = 25–49%, 3 = 50–74%, and 4 = 75–100%) and Thickness (1 = light, pink to light red, 2 = medium, red). Each tooth’s calculus was scored based on coverage using the following numerical scale: Coverage (0 = no calculus detected, 1 = 1–24%, 2 = 25–49%, 3 = 50–74%, and 4 = 75–100%). Each tooth’s gingivitis score was measured based on a modified Loe & Silness method (34) based on the following metrics: 0 = normal gingiva, 1 = moderate inflammation and redness, but no bleeding on probing, 2 = moderate inflammation with severe redness and bleeding on probing, and 3 = severe inflammation and redness, edema, ulceration, and spontaneous bleeding. The mean whole-mouth plaque, calculus, and gingivitis scores were calculated by averaging the total tooth scores for each animal.

#### 2.2.3 Collection of subgingival plaque samples for microbiome analysis

For each subject, subgingival plaque samples were collected from the healthiest and unhealthiest teeth on day 28 after grading the whole mouth. Healthy and unhealthy teeth were characterized based on individual tooth gingivitis scores. A gingivitis score of “0 = normal gingiva” was considered healthy, while a gingivitis score of ≥1 was considered unhealthy. If the subject did not have an unhealthy tooth, then subgingival plaque samples were collected from two different healthy teeth.

### 2.3 Microbiome sequencing and processing

Total DNA was extracted from frozen subgingival plaque samples using the Qiagen DNeasy PowerBiofilm Kit (Qiagen, Germantown, MD, USA), following the manufacturer’s instructions with the addition of a lysozyme step to improve efficiency. PCR amplification was performed with primer pairs that spanned the V1-V3 hypervariable regions of the 16S rRNA gene, along with the Illumina® Nextera XT Index Kit v2 Set A (96 indexes, 384 samples) under the following conditions in a C1000 Touch thermal cycler (Bio-Rad, Hercules, CA, USA): 25 cycles of 95 °C for 30 s, 58 °C for 30 s and 72 °C for 45 s, followed by 72 °C for 5 min. Amplicon quality was determined with the Agilent DNA 7500 Kit on the 2,100 Bioanalyzer. Amplicon sequencing was performed following the Illumina 16S metagenomic sequencing library preparation protocol (Part # 15044223 Rev. B) using the MiSeq v3 reagent cartridge kit and paired-end sequencing for 2/300 cycles. For each MiSeq run, a mock community sample was included as a positive control, and water was included as a negative control, to monitor the sequence run parameters and bioinformatics processing. Both the positive and negative control samples were processed identically to the subgingival DNA samples throughout the entire sequence processing protocol. Sequence runs with a quality score (Q30) of above 70% were processed for further analysis. The sequences were de-multiplexed to obtain FASTQ files. The FASTQ sequence files were processed into contigs from pairs of reads, and chimeras were removed using the standard parameters of mothur, version 1.39.5 (37). All retained sequences were aligned to the Human Oral Microbiome reference (HOMD) database (v15.11). Chimeras were identified using the UCHIME method within mothur and excluded from further processing (38). The sequences were then classified against the HOMD database using the naïve Bayesian classifier (39) within mothur with a minimum confidence threshold of 80% for each assignment.

### 2.4 Statistical analyses

Tooth subgingival plaque microbiome data were analyzed at the phylum and species levels. Only Operational Taxonomic Units (OTUs) at the species level that were present in at least 70% of all the samples were considered in the statistical analysis. Individual OTU counts were analyzed using negative binomial mixed models (40) to study the effects of age, time between the last and current dental prophylaxis, the number of diets fed to the dog 10 days before the dental examination, and the tooth gingivitis score. All *p*-values were adjusted for false discovery rate (FDR) using the Benjamini–Hochberg procedure (41). Principal coordinate analysis plots based on the Manhattan distance were made to visualize the proximity of the subgingival microbial compositions. All statistical microbiome analyses were carried out in R-4.2.3 (42).

### 2.5 Predictive model development

**Figure 1:**
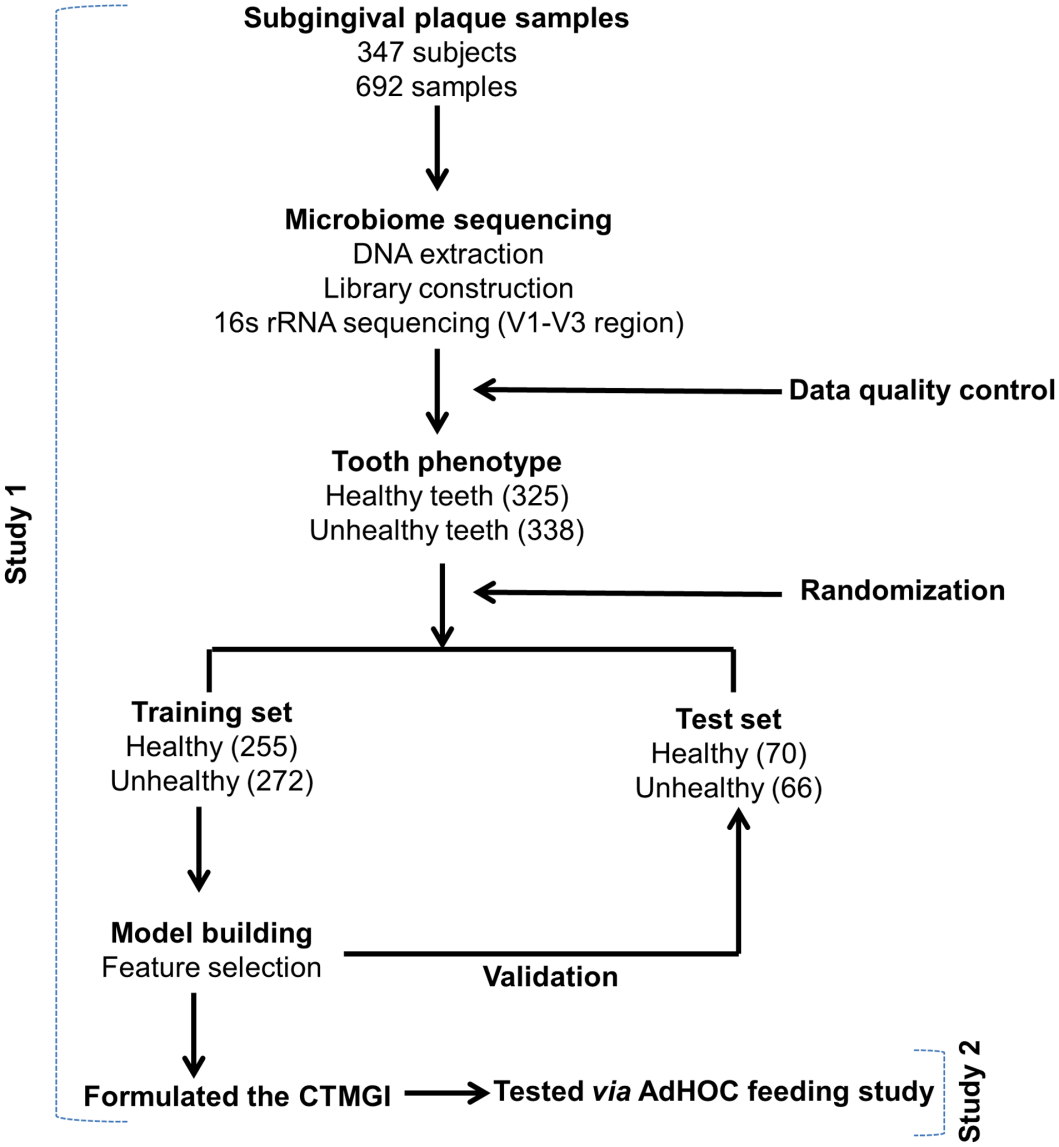
Flow diagram illustrating the schematics used to analyze the study data sets. CTMGI, Canine Tooth Microbiome Gingival Index.

Of the 90 OTUs identified as significant by the negative binomial mixed models, 54 OTUs (Supplementary Table S1) were chosen for inclusion in the classification modeling. This selection was based on a combination of factors, including their reported presence in human and canine literature, in addition to their abundance levels within this cross-sectional study. In addition, we also included other variables in the classification modelling such as the sum of OTUs represented as early colonizers (43), the sum of OTUs represented as late colonizers (Supplementary Table S2), and three phyla (Actinobacteria, Proteobacteria, and Spirochaetes), eleven blood chemistry variables, 13 disease co-morbid conditions and age. Classification modeling was performed in Dataiku, as illustrated in Figure 1. The cross-sectional study cohort data were randomly split 80/20 between the training and test sets. The training set was used for model training, and 5-fold cross-validation was used for hyperparameter tuning. The test set was used to evaluate the model’s performance. Random forest (RF), logistic regression, XGBoost, and LightGBM algorithms were used to find the model with the best performance based on the ROC AUC metric. Of these four classification models, random forest was the model with the best performance, with an ROC AUC of 74.5% (0.745), an accuracy of 74% (0.743), and a precision of 73% (0.737). This was followed by a logistic regression model, with an ROC AUC of 74% (0.74), an accuracy of 67.6% (0.676), and a precision of 72.2% (0.722) (Table 1). The top 20 features derived from the random forest model based on their VIP scores are shown in Supplementary Figure 2. These features include early colonizers, early-to-late colonizers ratio, phylum actinobacteria, phylum proteobacteria, and OTUs such as *Escherichia coli, Moraxella osloensis, Prevotella intermedia,* and *Neisseria weaveri*. Similarly, the top seven features derived from the logistic regression model based on their coefficient scores are listed in Supplementary Figure 3. These features include early colonizers, phylum actinobacteria, phylum proteobacteria, and OTUs such as *Mollicutes* sp. *HMT_906* and *Fastidiosipila sanguinis.*

**Table 1:** Performance metrics of various machine learning models used in this study to identify the significant features associated with healthy and gingivitis-affected teeth.

Model	ROC AUC	Accuracy	F1 score
Random forest	0.745	0.743	0.737
Logistic regression	0.74	0.676	0.722
XGBoost	0.701	0.691	0.700
LightGBM	0.700	0.699	0.701

## 3 Results

### 3.1 Meta-analysis of cross-sectional study cohort

In total, 692 subgingival plaque samples were collected from 347 dogs, including 168 males and 179 females. These dogs varied in reproductive status, and their ages ranged from 1.9 to 14.8 years (mean: 8.1 ± 3.9 years). The dental examination intervals for the dogs recruited in this study typically spanned 0.3–1.9 years, with the single exception of a 3.1-year-old subject undergoing its first annual examination during the study. Blood CBC and serum chemistry analysis were completed on all dogs in close proximity to their dental examination: 288 samples were taken on the day of the procedure, 50 samples were collected within 10 days of the procedure, and the remaining 9 samples were collected within 22 days of the date of the dental examination. Of the blood CBC and serum chemistry analytes, 11 were included to reflect the immune status of the subjects. The following are the mean ± SD values for the actual blood analytes and their calculated ratios: neutrophils 3.4 ± 1.42 K/μL, lymphocytes 1.41 ± 0.47 K/μL, monocytes 0.29 ± 0.14 K/μL, eosinophils 0.2 ± 0.14 K/μL, basophils 0.01 ± 0.01 K/μL, and platelets 284.7 ± 134.3 K/μL, Neutrophil-to-Lymphocyte Ratio (NLR) 2.56 ± 1.06, Platelet-to-Lymphocyte Ratio (PLR) 223.1 ± 130.3, Monocyte-to-Lymphocyte Ratio (MLR) 0.22 ± 0.09, Eosinophil-to-Lymphocyte Ratio (ELR), 0.15 ± 0.11, and the Systemic Immune-Inflammation Index (44) was 751.7 ± 539.7.

All 347 dogs were categorized based on the food they ate within 10 days of the dental examination. The categories were: a single food (34.6%), two foods (15.9%), and >2 foods (ranging between 2 and 12 distinct foods). Similarly, the underlying co-morbid disease conditions in the cohort included clinically healthy dogs (*n* = 107), a single co-morbid disease condition (*n* = 102), and the remaining dogs (*n* = 138) that had between 2 and 7 co-morbid disease conditions. These co-morbid conditions included musculoskeletal disorders (*n* = 118), skin disorders (*n* = 131), oral abnormalities (*n* = 70), and gastrointestinal issues (*n* = 35). Both food consumption and co-morbid disease condition data were included as numerical data in the analyses.

### 3.2 Relationship between whole-mouth scores vs. individual tooth gingivitis scores

**Figure 2:**
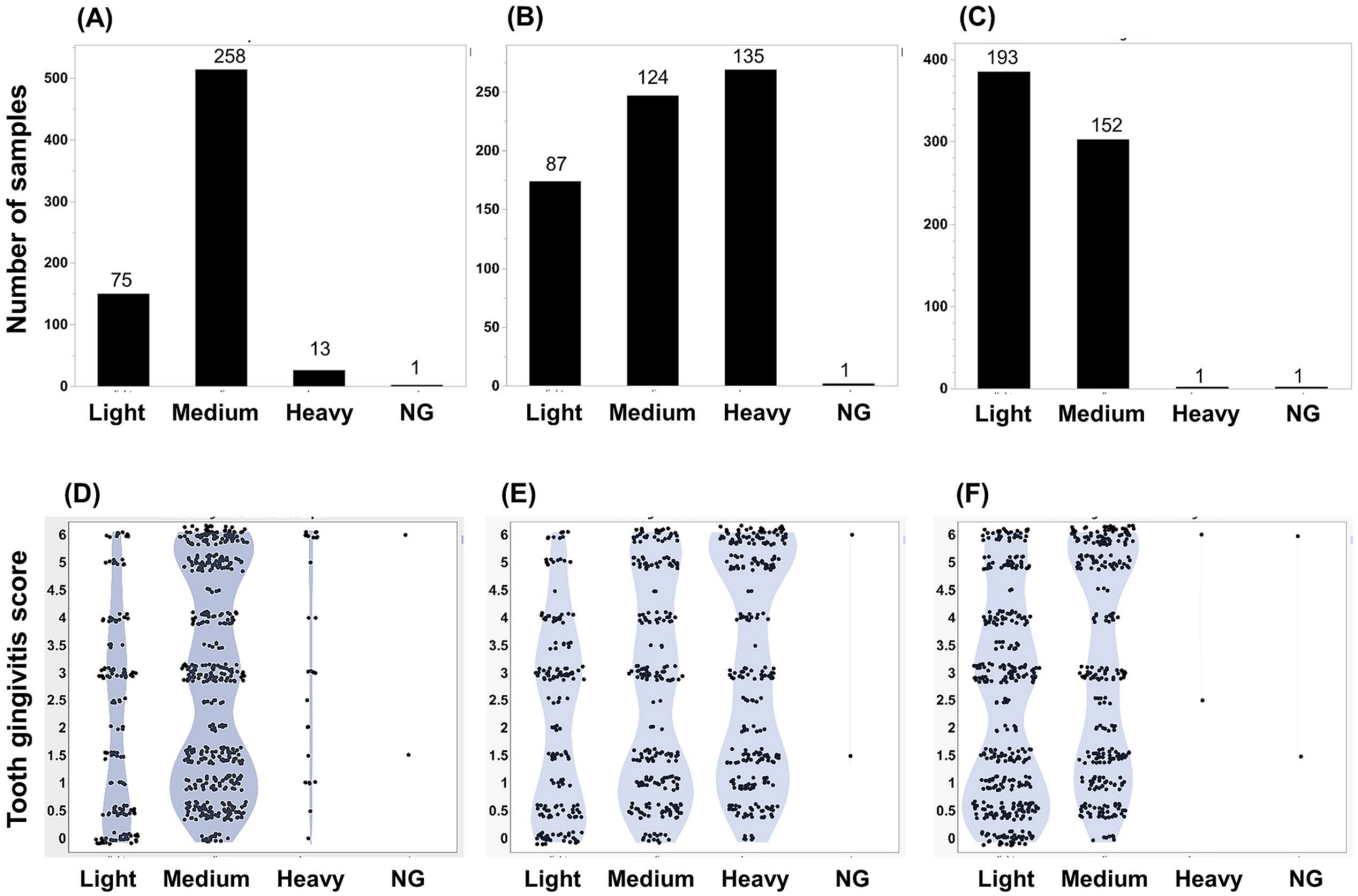
Distribution of subgingival plaque samples by whole-mouth **(A)** plaque, **(B)** calculus, and **(C)** gingivitis scores, and individual tooth gingivitis scores plotted against whole-mouth **(D)** plaque, **(E)** calculus, and **(F)** gingivitis scores. The numbers represented on the bars indicate the number of canine subjects in each grade. NG: Not graded.

Whole-mouth scores for calculus, plaque, and gingivitis were evaluated for 347 dogs (Figures 2A–C). Whole mouth scores were categorized as light, medium, or heavy, as described above. The cohort showed a “medium” whole-mouth plaque score, representing the highest number of samples (*n* = 514, 74.27% derived from 258 dogs), followed by “light” (*n* = 75, 21.67% derived from 75 dogs). In contrast, the cohort showed almost similar representation across the grading for whole-mouth calculus scores: light (*n* = 174, 25.14% derived from 87 dogs); medium (*n* = 124, 35.69% derived from 124 dogs), and heavy (*n* = 135, 38.87% derived from 135 dogs). Whole-mouth gingivitis scores showed a higher number of samples in the “light” (*n* = 385, 55.63% derived from 193 dogs) category, followed by the “medium” (*n* = 303, 43.78% derived from 152 dogs) category. Overall, these whole-mouth scores suggest that the cohort represents an early gingivitis (reversible) condition rather than periodontitis, an irreversible condition.

Furthermore, we plotted the individual tooth gingivitis scores (representing two teeth) against categorical whole-mouth plaque, calculus, and gingivitis scores (representing the entirety of the oral cavity) to understand their relationship (Figures 2D–F). These plots did not show any specific trends, and surprisingly, the tooth gingivitis scores (0–6) were distributed almost evenly across the “light” and “medium” categories of the whole-mouth plaque, calculus, and gingivitis scores. Interestingly, the presence of higher tooth gingivitis scores in the “light” category of whole-mouth plaque and calculus suggests that the overall building of plaque and calculus might not be the primary causative factor for gingivitis in this cross-sectional study cohort.

### 3.3 Samples and microbiome sequence quality

Among the 692 subgingival plaque samples, 663 samples were included in the analyses after removing the 29 samples with low read counts (<5 K) prior to the analyses. The total number of paired quality sequence reads included in the analyses was 53,773,786, and the mean ± SD read count per sample was 80,984 ± 35,813. These assembled sequences were assigned to 590 OTUs. Applying the filtration criterion that OTUs must be present in at least 70% of all samples yielded 113 species. These 113 species represented over 90% of the reads per sample, with the exception of only 13 samples, where they accounted for 70–89% of the reads.

### 3.4 Subgingival microbiome profile shows trending by individual tooth gingivitis scores

**Figure 3:**
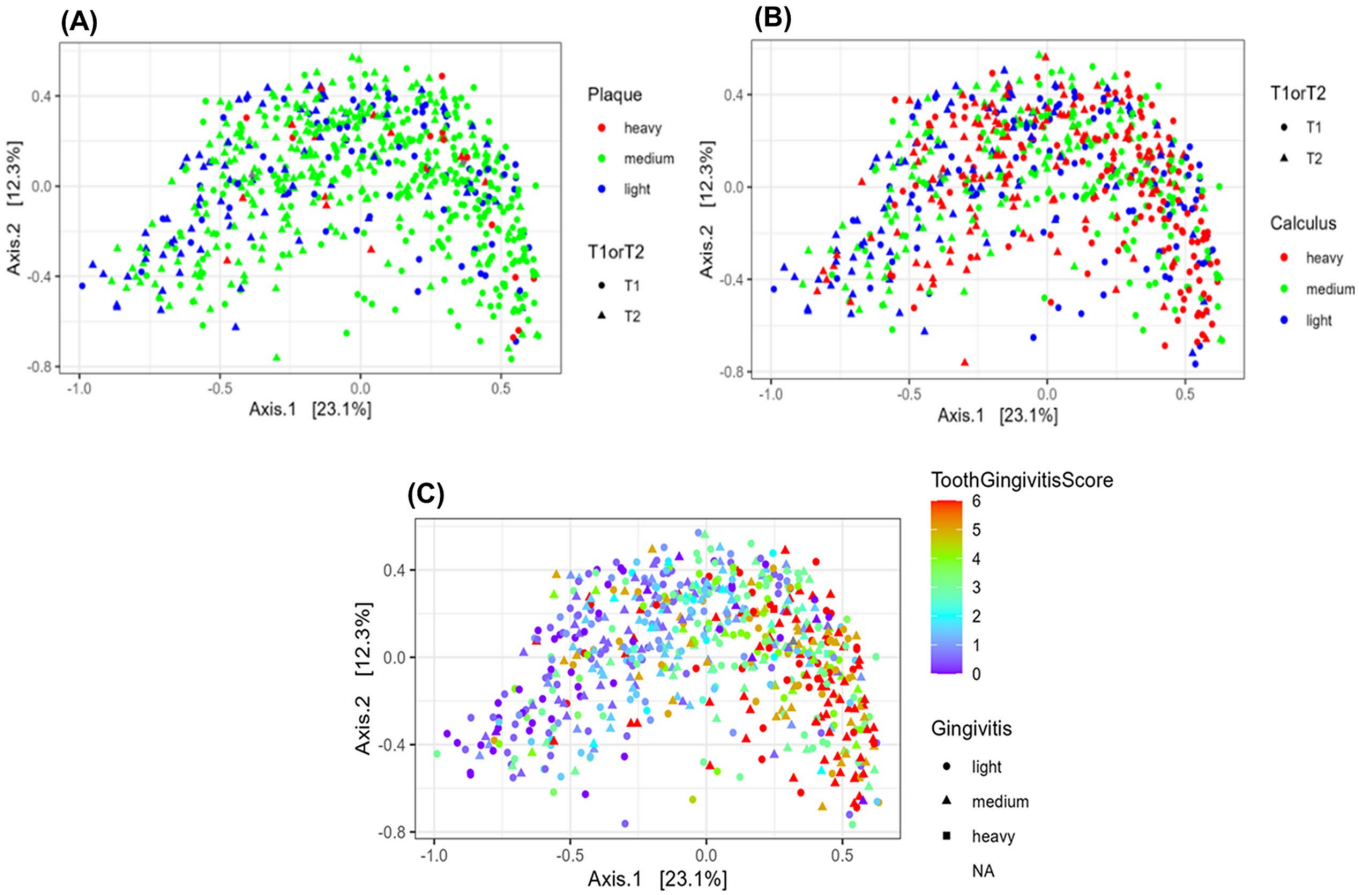
Principal coordinate analyses of subgingival microbiome composition: **(A)** Whole-mouth plaque scores, **(B)** whole-mouth calculus scores, **(C)** whole-mouth gingivitis and individual tooth gingivitis scores.

**Figure 4:**
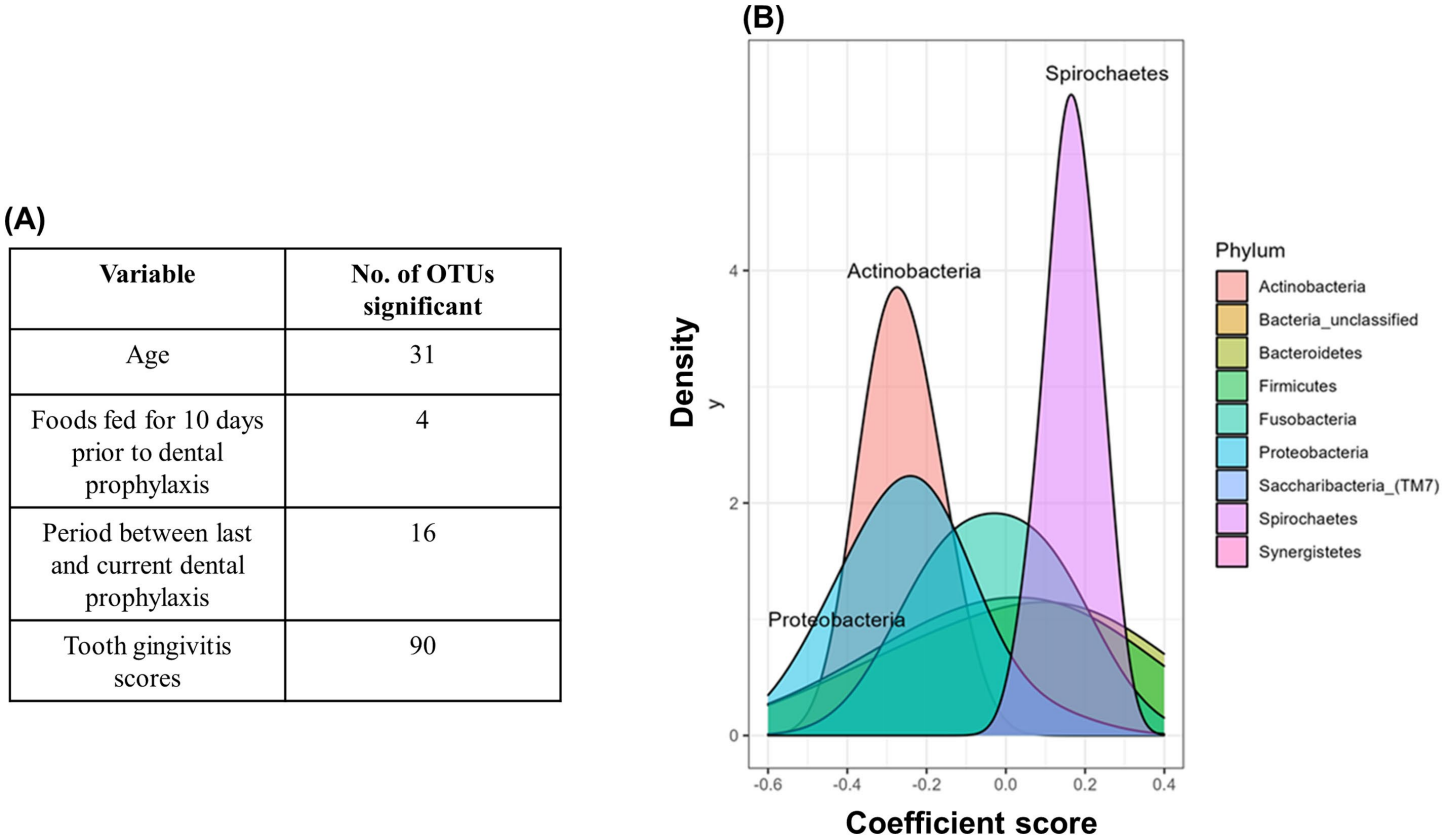
**(A)** Negative binomial mixed model analysis reveals the number of OTUs significantly associated with the variables tested. **(B)** A density plot of the 90 OTUs shows that the significance of tooth gingivitis scores is represented at the phylum level. The “*x*” axis represents the coefficient scores associated with tooth gingivitis scores, and the “*y*” axis represents phylum abundance as density. Negative and positive coefficient values on the “*x*” axis indicate an association with low and high tooth gingivitis scores (0–6), respectively.

Principal coordinate analyses (PCoA) based on the Manhattan distance metrics were used to examine the relationship trends between the subgingival microbiome composition and whole-mouth plaque, calculus, and gingivitis scores, along with individual tooth gingivitis scores (Figures 3A–C). Overall, the first component explained 23.1% of the variability in the OTU proportions, and the second component explained 12.3%. PCoA plots did not reveal any trends or separations based on whole-mouth plaque, calculus, or gingivitis scores. However, there was a trend observed based on individual tooth gingivitis scores (Figure 3C) regardless of whole-mouth gingivitis scores. Furthermore, we employed a negative binomial mixed model to study the effects of age, the number of foods fed 10 days prior to the dental examination/prophylaxis, the period between the last and current dental examinations/prophylaxis, and the tooth gingivitis score to identify the significance of individual OTUs. Of these factors, tooth gingivitis scores impacted the highest number of significant OTUs (*n* = 90), followed by age (*n* = 31) (Figure 4A). The list of significant OTUs associated with age, the number of foods fed 10 days prior to dental prophylaxis, the period between the last and current dental prophylaxis, and the tooth gingivitis score are listed in Supplementary Tables S3–S6. These 90 significant OTUs represented 9 phyla, with the density plot showing that the phylum Spirochaetes was positively associated with higher tooth gingivitis scores, while the phyla Actinobacteria and Proteobacteria were negatively associated with higher tooth gingivitis scores (Figure 4B; Supplementary Table S7). OTUs positively associated with higher tooth gingivitis scores include *Treponema* sp., *Porphyromonas* sp., *Fusobacterium nucleatum sub*sp. *Vincentii, Tannerella forsythia*, and *Prevotella intermedia*, which have previously been described as late colonizers (red and orange complex bacterial species) (22, 45) (Supplementary Table S6). Similarly, OTUs negatively associated with higher gingivitis scores include *Streptococcus* sp., *Bergeyella* sp., *Moraxella* sp.*,* and *Capnocytophaga* sp. *Escherichia coli* and *Neisseria* sp., which have been previously described as early colonizers (22, 45).

### 3.5 Categorizing individual tooth gingivitis scores into a binary variable (healthy vs. unhealthy) for classification modeling

**Figure 5:**
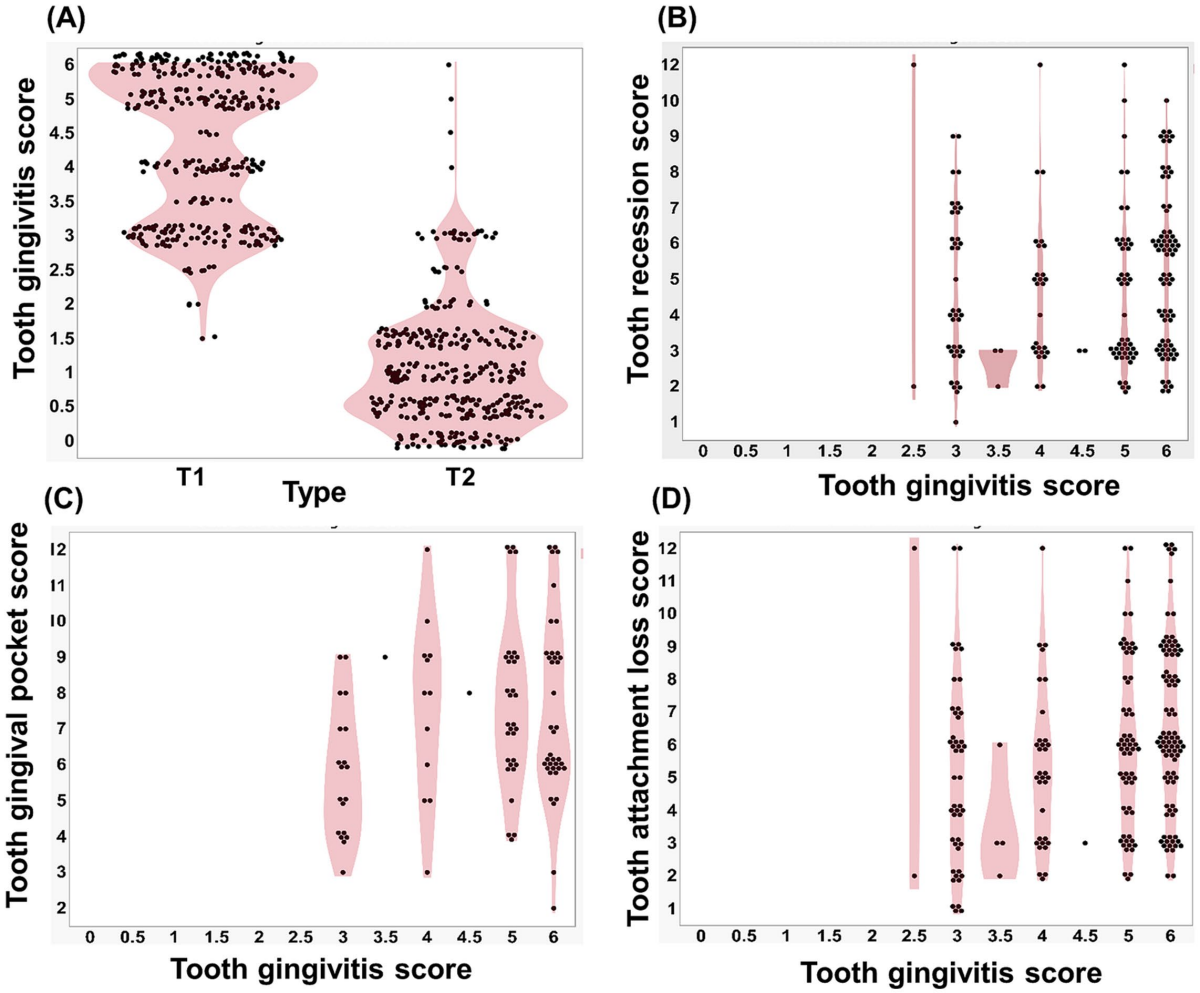
Characterization of the tooth from which the subgingival plaque samples were collected for this study. **(A)** Distribution of tooth gingival scores: T1 (unhealthy) and T2 (healthy). **(B)** Distribution of tooth recession scores (mm) vs. tooth gingival scores. **(C)** Distribution of gingival pocket scores (mm) vs. tooth gingival scores. **(D)** Distribution of tooth attachment loss scores (mm) vs. tooth gingival scores.

Individual tooth gingivitis scores were categorized as “T1_unhealthy” (mean ± SD 4.5 ± 1.21) or “T2_healthy” (mean ± SD 0.94 ± 0.66) based on the individual tooth gingivitis score (≥3: unhealthy; <3: healthy) (Figure 5A). The unhealthy category showed poorer clinical outcomes, such as tooth recession (Figure 5B), pocket score (Figure 5C), and attachment loss score (Figure 5D). The distribution plots showed that the clinically relevant observations of tooth recession, pockets, and attachment loss were made with a tooth gingivitis score ≥3. Conversely, clinically relevant observations of tooth recession, pockets, and attachment loss were not made with tooth gingivitis scores <3.

### 3.6 Development of the Canine Tooth Microbiome Gingival Index (CTMGI)

To develop the CTMGI, we used the 22 most important and significant features from the best-performing (random forest) and the second best-performing (logistic regression) models, excluding their overlapping features (Table 2, Supplementary Figures S2, S3). These features were determined to be either “positive” or “negative” based on their effect on the outcome of the tooth gingivitis condition. The formula to derive the CTMGI is as follows:

$$\text{CTMGI}=\log[\begin{array}{l} \text{average}(\text{positive features})/\\ \text{average}(\text{negative features}) \end{array}]$$

**Table 2:** The top 22 features derived from the top two performance models and their association with healthy teeth used to calculate the Canine Tooth Microbiome Gingival Index (CTMGI).

OTU ID	Association	Features	Model
Early colonizers	Positive	Early colonizers	RF, LR
ra_Actinobacteria	Positive	Relative abundance_Phylum Actinobacteria	RF, LR
ra_Proteobacteria	Positive	Relative abundance_Phylum Proteobacteria	RF, LR
Otu012	Positive	Bacteria; Proteobacteria; Gammaproteobacteria; Pseudomonadales; Moraxellaceae; Moraxella; osloensis	RF
Otu013	Positive	Bacteria_Proteobacteria_Gammaproteobacteria_Pseudomonadales _Moraxellaceae_Acinetobacter_johnsonii	RF
Otu014	Positive	Bacteria; Proteobacteria; Gammaproteobacteria; Pseudomonadales; Moraxellaceae; Moraxellaceae_unclassified; Moraxellaceae_unclassified	RF
Otu017	Positive	Bacteria; Proteobacteria; Gammaproteobacteria; Pasteurellales; Pasteurellaceae; Haemophilus; Haemophilus_unclassified	RF
Otu049	Positive	Bacteria; Bacteroidetes; Flavobacteriia; Flavobacteriales; Flavobacteriaceae; Bergeyella; sp._HMT_422	RF
Otu054	Positive	Bacteria; Firmicutes; Bacilli; Lactobacillales; Streptococcaceae; Streptococcus; Streptococcus_unclassified	RF
Otu088	Positive	Bacteria_Firmicutes_Erysipelotrichia_Erysipelotrichales_Erysipelotrichaceae _Solobacterium_moorei	RF
Late colonizers	Negative	Late colonizers	LR
Otu006	Negative	Bacteria_Spirochaetes_Spirochaetia_Spirochaetales_Spirochaetaceae _Treponema_denticola	RF
Otu011	Negative	Bacteria_Fusobacteria_Fusobacteriia_Fusobacteriales_Fusobacteriaceae _Fusobacterium_nucleatum_subsp._vincentii	RF
Otu039	Negative	Bacteria_Spirochaetes_Spirochaetia_Spirochaetales_Spirochaetaceae _Treponema_sp._HMT_490	RF
Otu046	Negative	Bacteria_Firmicutes_Clostridia_Clostridiales_Ruminococcaceae _Ruminococcaceae_unclassified_Ruminococcaceae_unclassified	RF
Otu050	Negative	Bacteria_Firmicutes_Mollicutes_Mollicutes_[O-2]_Mollicutes _[F-2]_Mollicutes_[G-2]_bacterium_HMT_906	LR
Otu058	Negative	Bacteria; Firmicutes; Clostridia; Clostridiales; Peptostreptococcaceae _[XI]; Peptostreptococcaceae_[XI][G-8]; bacterium_HMT_382	RF, LR
Otu060	Negative	Bacteria; Spirochaetes; Spirochaetia; Spirochaetales; Spirochaetaceae; Treponema; sp._HMT_239	RF
Otu065	Negative	Bacteria; Proteobacteria; Gammaproteobacteria; Enterobacterales; Enterobacteriaceae; Escherichia; coli	RF
Otu071	Negative	Bacteria_Firmicutes_Clostridia_Clostridiales_Ruminococcaceae _Fastidiosipila_sanguinis	LR
Otu083	Negative	Bacteria_Spirochaetes_Spirochaetia_Spirochaetales_Spirochaetaceae _Treponema_sp._HMT_238	RF
Otu093	Negative	Bacteria_Firmicutes_Mollicutes_Mycoplasmatales_Mycoplasmataceae _Mycoplasma_lipophilum	RF

RF, Random forest; LR, Logistic regression.

**Figure 6:**
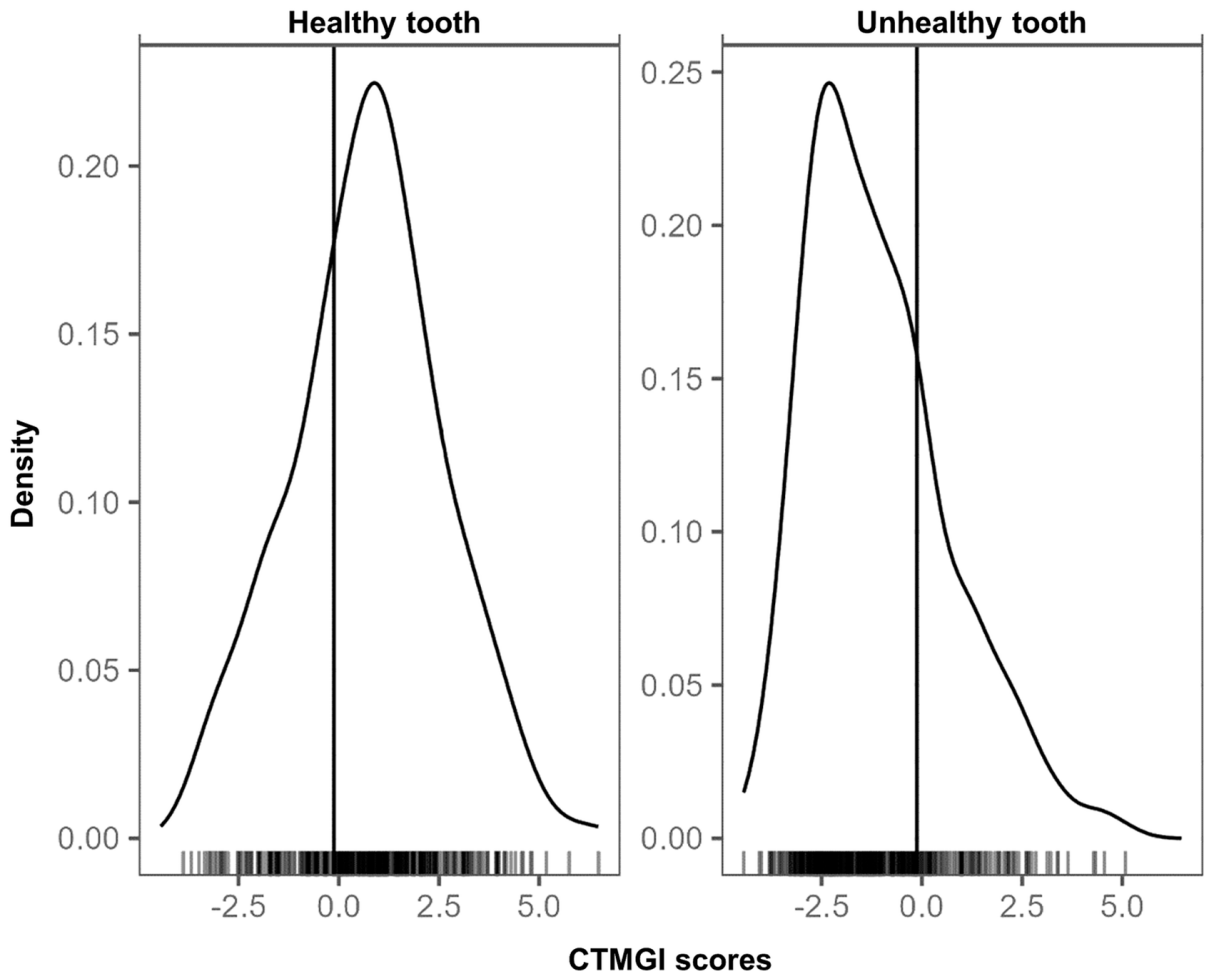
Density plot visualizing the distribution of calculated CTMGI scores for healthy and unhealthy teeth. The vertical line indicates the optimal threshold of −0.12, with an ROC AUC of 0.761, sensitivity of 0.701, and specificity of 0.752. Values > − 0.12 are considered healthy, while values less than that are considered unhealthy. CTMGI, Canine Tooth Microbiome Gingival Index.

The classification cutoff for CTMGI was set at −0.12 to maximize the sum of sensitivity and specificity (Figure 6). A tooth gingivitis condition would be classified as “healthy” if the CTMGI > − 0.12, and “unhealthy” otherwise. It provided an ROC AUC of 0.761, sensitivity of 0.701, and specificity of 0.752. The CTMGI offers a simpler approach to classify the tooth gingivitis condition as a single number compared to the classification models with similar performance.

### 3.7 Real-time validation of the CTMGI by a controlled nutritional intervention study

**Figure 7:**
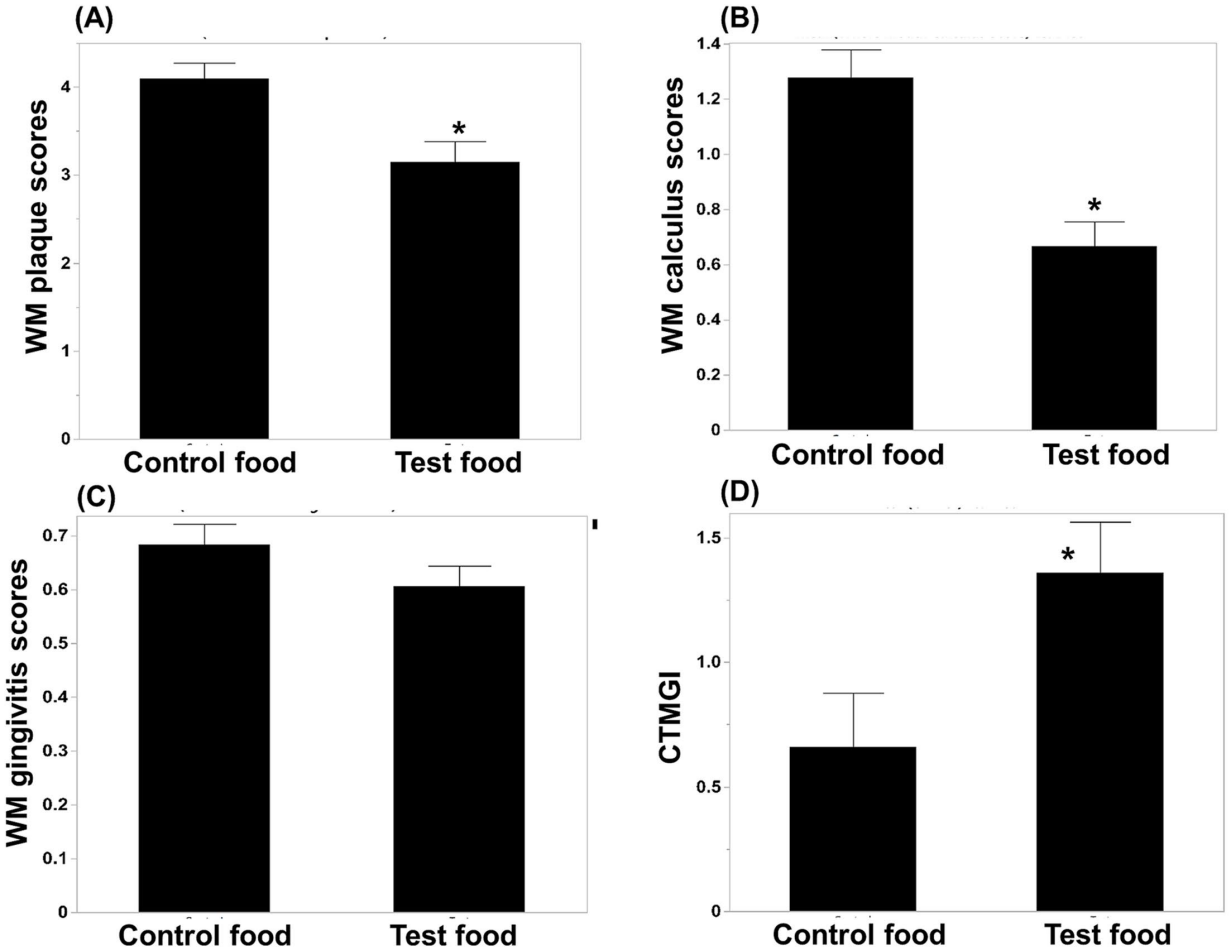
**(A)** Whole-mouth plaque, **(B)** calculus, **(C)** gingivitis, and **(D)** CTMGI scores of dogs fed the control and test foods used in this study. Asterisks indicate the significance (*p* < 0.05) by *t*-test. The control food has no known oral health benefits, and the test food has proven oral health benefits *via* a mechanical effect.

We determined the applicability and feasibility of the CTMGI on microbiome data derived from our controlled nutritional intervention study (see details in Materials and Methods, 2.2 Study 2 for details). This parallel-arm study design included two sets of small-breed, healthy adult dogs, with one set of dogs fed control food (no known oral health benefits), and another set of dogs fed test food (with proven oral health benefits through its mechanical effect) for a period of 28 days. All dogs underwent dental prophylaxis at the baseline. Dental grading was performed by a trained dental grader, followed by the collection of subgingival plaque samples for microbiome analyses. Whole-mouth plaque and calculus scores showed a significant reduction with the test food compared to the control food (Figures 7A,B); however, a numerical decrease was observed in whole-mouth gingivitis scores due to the 28-day study period (Figure 7C). We also analyzed the subgingival plaque microbiome data after performing quality control. The microbial OTU abundance data were transformed into proportions, and we selected specifically the 22 features to calculate the CTMGI. The CTMGI score was significantly higher (1.36) in the dogs fed the test food than in the dogs fed the control food (0.66), suggesting that the test food shifts or maintains the subgingival microbiome toward a healthier tooth gingivitis condition (Figure 7D).

## 4 Discussion

The composition of the oral cavity microbiome is intricate, exhibiting both overlapping and distinct features depending on the specific oral niche (17, 18, 20, 30). Notably, the subgingival microbiome composition is a critical factor in triggering gingival inflammation (17, 18, 20). Gingivitis is a reversible condition; however, failure to treat it promptly can lead to periodontitis, which is an irreversible disease (46). While gingivitis is a risk factor for periodontitis, not all gingivitis-affected tooth sites will progress to this more severe condition (47). Furthermore, diagnosis using the microbiome is essential, especially considering that visual assessment alone is only successful in diagnosing 9–20% of dogs (5, 48). Historically, the comprehensive reporting of microbiome data—simply listing microbial species and their abundance levels—has limited its clinical applicability. Therefore, there is a clear need for clinically relevant and easily interpretable summary statistics derived from these complex microbiome profiles.

To the best of our knowledge, this study represents a significant advancement, as it is the first microbiome-based diagnostic measure for dogs, the Canine Tooth Microbiome Gingival Index (CTMGI). This index was derived using a holistic approach that analyzed the entire microbial community using machine learning models to identify 22 significant features from a large cohort of 692 subgingival plaque samples collected from 347 canine subjects. The CTMGI was formulated using the 22 features, including 18 specific microbial species, two phyla (Actinobacteria and Proteobacteria), early colonizers (a group of 12 species), and late colonizers (a group of 21 species) (Table 2). These early and late colonizer species were selected based on published reports of the human oral microbiome (49) (Supplementary Table S2). Specifically, the late colonizer group encompasses orange and red complex bacterial species, such as *Prevotella intermedia, Fusobacterium nucleatum sub*sp. *Vincentii, Campylobacter rectus, Porphyromonas gingivalis, Treponema denticola,* and *Tannerella forsythia.* Some of these species were recently identified in canine supragingival plaque microbiome samples analyzed using metagenomics technology rather than 16S amplicon sequencing technology (50), with many undercharacterized species. Furthermore, this study pooled supragingival plaque samples to perform metagenomics sequencing technology for broader coverage, rather than profiling individual tooth plaque samples, as previously described. The identification of similar late colonizer group species in this study using 16S sequencing technology reinforces the relevance of the species incorporated into the CTMGI development.

This group of species identified as late colonizers is specifically highlighted because of its crucial role in initiating gingival inflammation. These species flourish in the anaerobic environment of the subgingival space, as previously reported (17), where anaerobic bacterial abundance is higher in subgingival plaque than in plaque collected from the gingival margin; this leads to an imbalance in the overall microbiome community that promotes their proliferation. This warrants our stepwise holistic approach for developing the CTMGI: first, by identifying significant bacterial OTUs associated with tooth gingivitis scores for the whole microbial community and two phyla using a negative binomial model. Then, those features were included along with other host variables (11 blood CBC variables and a comorbidity condition proxy for host immune status) in machine learning models. We included other host variables in the machine learning models along with significant bacterial OTUs to test the “IMPEDE” hypothesis, which highlights the role of the host’s immune response in maintaining microbial balance. Failure of this balance allows specific pathogenic species to dominate and drive disease progression (14).

Employing machine learning (ML) models for disease prediction, particularly in relation to the microbiome (51), is increasingly being evaluated. However, interpreting these models for clinical translation remains a challenge compared to a simple, single index value. Clinicians can easily interpret a single index value by comparing a patient’s score to a threshold to determine a healthy *vs.* unhealthy status. In this study, we addressed this challenge by using significant features derived from our top-performing ML models to calculate the CTMGI based on the positive or negative relationship of these features with gingival scores. It classifies teeth as healthy *vs.* unhealthy. We established the CTMGI cutoff score at −0.12, where scores greater than −0.12 are considered indicative of a healthy tooth (indicating less to no risk of developing gingivitis), while scores at or below this value are considered to indicate an unhealthy tooth (indicating a risk of developing gingivitis). This cutoff yielded a robust diagnostic performance with an ROC AUC of 0.761, a sensitivity of 0.701, and a specificity of 0.752 (Figure 6).

Two previous studies have developed molecular diagnostic tools based on the canine oral microbiome to predict periodontal disease (28, 52). Both approaches utilized qPCR assays that targeted specific microbial species to distinguish between a “reversible group” (healthy, gingivitis, and early periodontitis) and an “irreversible group” (periodontitis). Kwon et al. (52) developed their diagnostic tool using qPCR to detect 11 microbial species. Ruparell et al. (28) initially developed an assay targeting 41 bacterial species. They found that the qPCR data for 30 of these species strongly correlated (*r* > 0.8) with high-throughput 454 pyrosequencing data using Pearson’s correlation, which dropped to 25 species using Spearman’s Rank correlation (*r* > 0.8). Ultimately, based on machine learning classification models, Ruparell et al. (28) proposed a molecular diagnostic tool that focused on only three targeted microbial species: one associated with periodontal health and two associated with periodontal disease. However, these three species have sensitivity ranging from 60.0 to 85.7%, and a specificity ranging from 27.5 to 80.0%. Additionally, this study classified the cohorts as reversible or irreversible categories, with reversible groups including healthy teeth, gingivitis, and early periodontitis.

We took this opportunity to make earlier predictions by analyzing a cohort of healthy dogs compared to those with gingivitis. Analyzing the subgingival plaque microbiome composition at the individual tooth level is critical due to the heterogeneous nature of the oral cavity and the fact that localized microbiome-induced inflammation impacts distant healthy gums in the oral cavity (53). Conversely, pooling samples from all teeth may dilute diagnostic accuracy, making a tooth-by-tooth analysis essential for a comprehensive understanding. Furthermore, this study successfully demonstrated the utility of the CTMGI through a nutritional intervention, as shown in Figure 7. Specifically, dogs fed the test food, which provided an oral health benefit, exhibited a CTMGI score of 1.36. This contrasts with the control group, whose food did not offer an oral health benefit. This finding aligns with the clinical assessment, which demonstrated a significant reduction in whole-mouth plaque and calculus scores in dogs consuming the test food compared to those fed the control food (Figures 7A,B). Overall, these results suggest that the CTMGI is sensitive to nutritional intervention and that the test food effectively shifts the oral microbiome toward a healthier state.

This study successfully created a single index score from the canine subgingival microbiome to assess gingival health status and validated its effectiveness *via* a nutritional intervention, but its practical application has limitations. The primary limitation is the reliance on subgingival plaque samples, which are difficult for pet owners to collect from dogs without professional assistance. Therefore, future research should aim to implement this index score using the supragingival plaque or saliva microbiomes to simplify the sample collection process and enhance the efficiency of its utility.

## 5 Conclusion

We developed and validated a quantitative, easy-to-interpret microbiome-based index for the assessment of canine gingival health. This tool enables veterinary clinicians to evaluate gingival health at the tooth level and proactively identify dogs at risk for periodontitis. Furthermore, we have shown that this index is effective in monitoring the success of nutritional interventions.

## Data Availability

The raw data supporting the conclusions of the article will be made available by the authors upon request. The microbiome raw sequence data presented in this study are deposited in the National Center for Biotechnology and information (NCBI) repository under the accession number PRJNA1442175.
