# Supplementary figures and images for: Canine Tooth Microbiome Gingival Index: a new microbiome-derived measure of gingival health validated by nutritional intervention
Source: Front Vet Sci. 2026 Jun 5;13:1839039. doi: 10.3389/fvets.2026.1839039 (PMC13280792; doi:10.3389/fvets.2026.1839039)

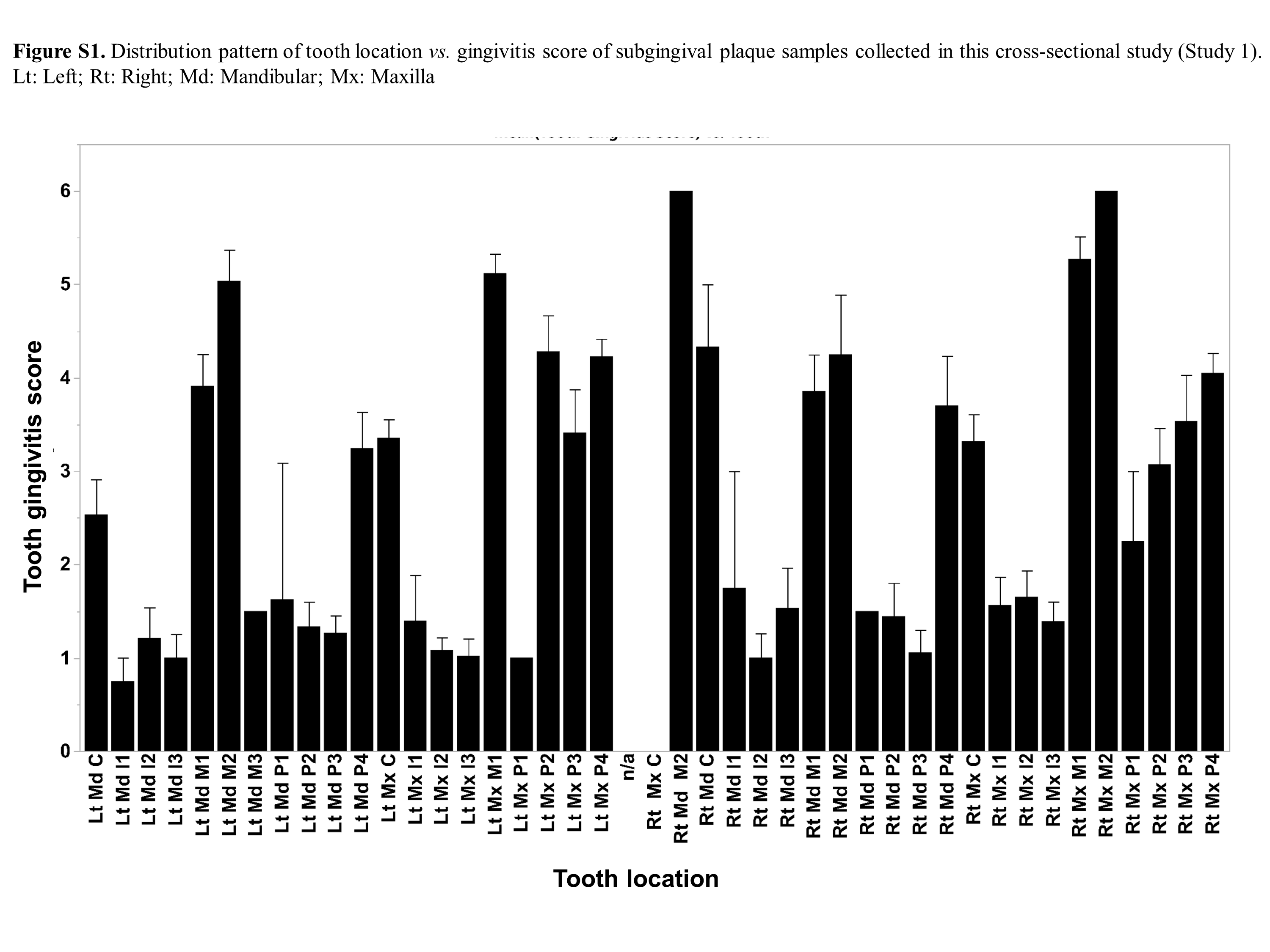

Supplement: Supplementary file 1 [file Figure_S1.tif]

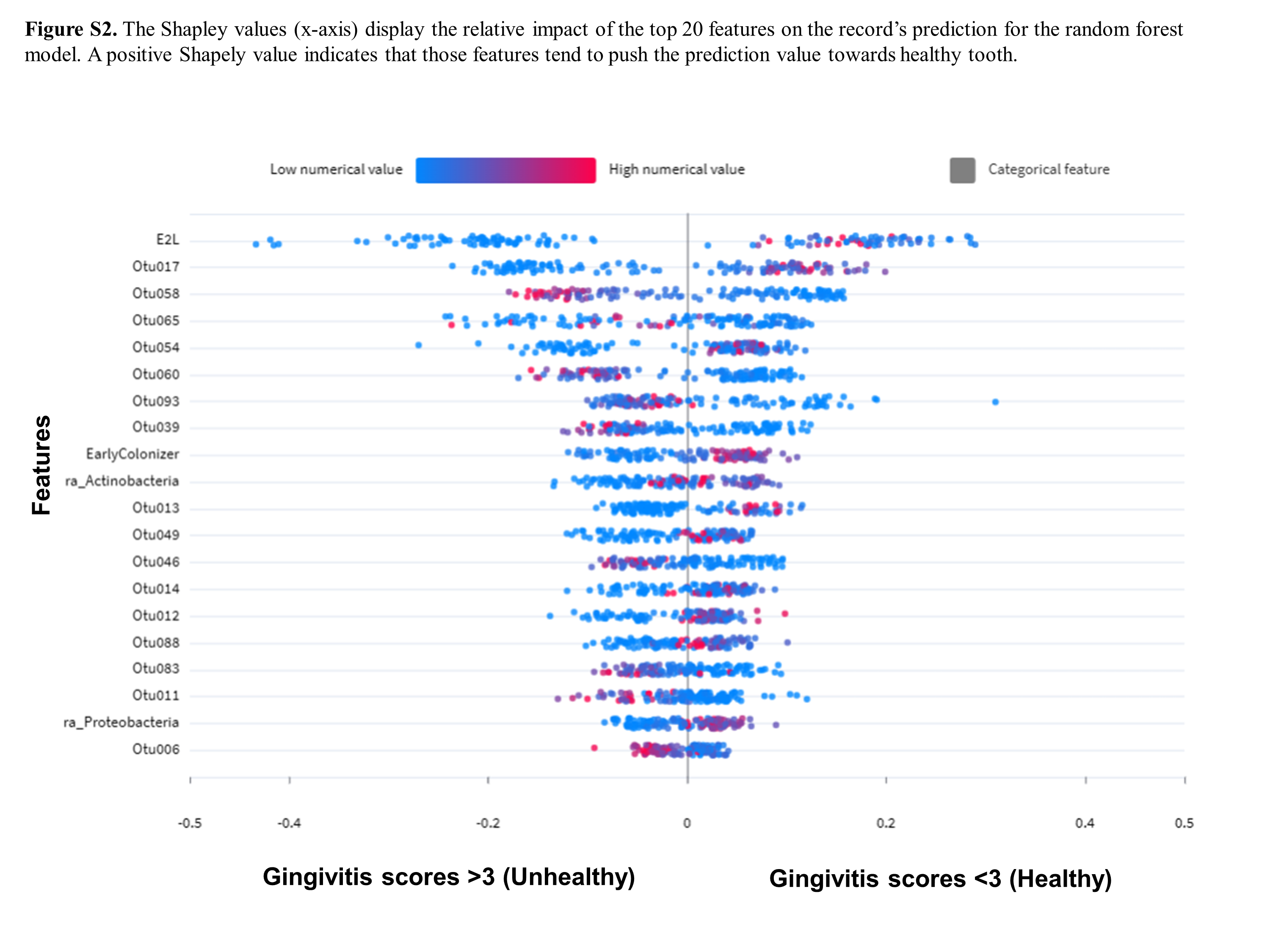

Supplement: Supplementary file 2 [file Figure_S2.tif]

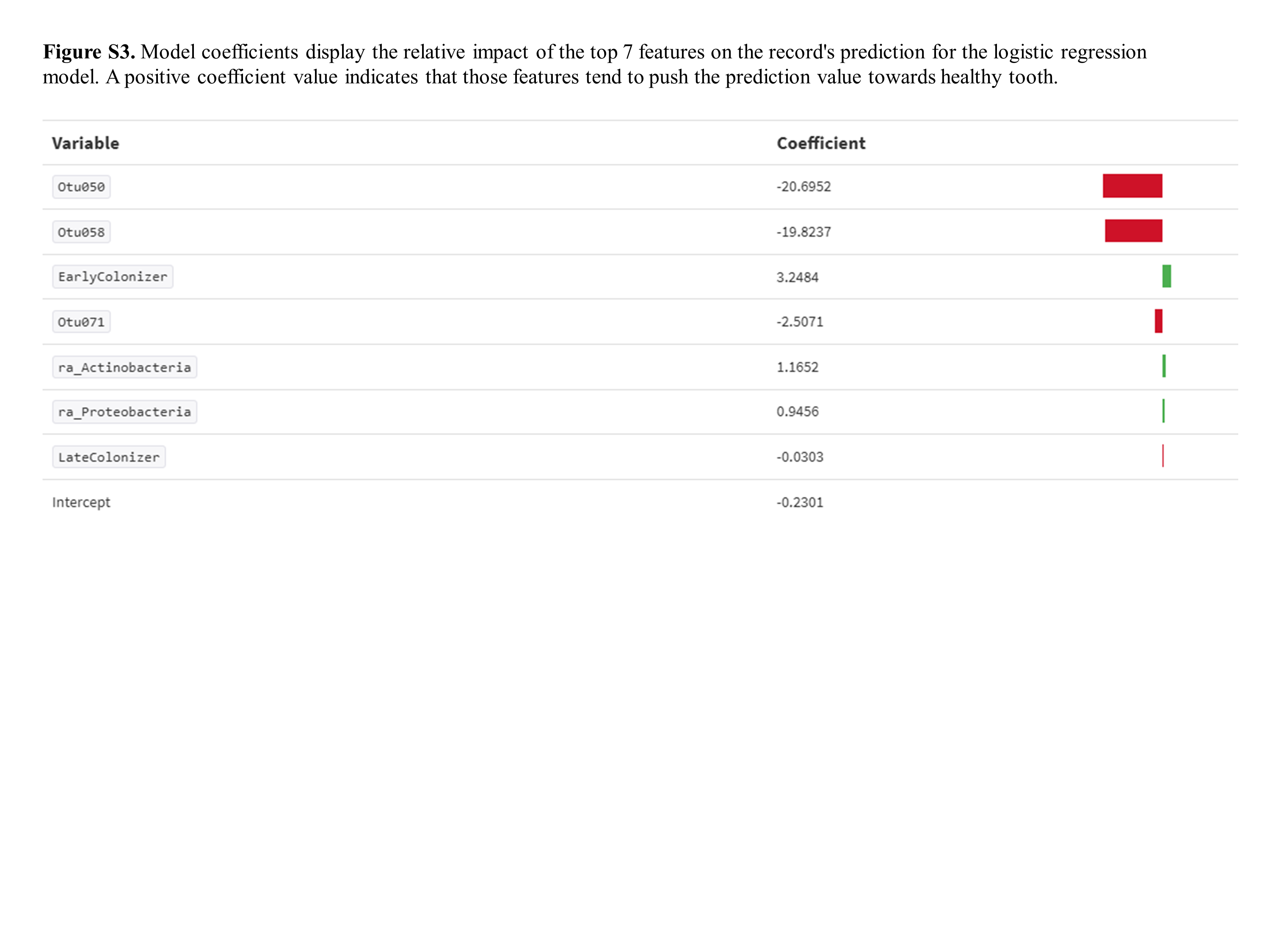

Supplement: Supplementary file 3 [file Figure_S3.tif]
